# Strain-dependent efficacy of pasteurized *Akkermansia muciniphila* in alleviating colitis reveals AKK2645 restores immune and microbial homeostasis

**DOI:** 10.3389/fnut.2026.1863368

**Published:** 2026-07-27

**Authors:** Hui Li, Zhili He, Minlei Zhao, Jie Yang, Yundan Bai, Jianguo Chen, Zengning Li

**Affiliations:** 1 College of Chemistry and Materials Science, Key Laboratory of Analytical Science and Technology of Hebei Province, and MOE Key Laboratory of Medicinal Chemistry and Molecular Diagnostics, Hebei University, Baoding, Hebei, China; 2 Beijing Youngen Technology Co. Ltd, Beijing, China; 3 Department of Infectious Diseases (Hepatology), Lishui Central Hospital, Lishui, Zhejiang, China; 4 Health Management Medical Center, Chengdu Integrated TCM and Western Medicine Hospital, Chengdu, Sichuan, China; 5 Department of Clinical Nutrition, The First Hospital of Hebei Medical University, Hebei Key Laboratory of Nutrition and Health, Shijiazhuang, Hebei, China

**Keywords:** AKK2645, gut microbiota, immunoregulation, pasteurized *Akkermansia muciniphila*, ulcerative colitis

## Abstract

Ulcerative colitis (UC) is a chronic inflammatory disease caused by intestinal immune dysregulation and microbial imbalance. Pasteurized *Akkermansia muciniphila* has attracted significant attention in recent research due to its anti-inflammatory properties and favorable safety profile. In this study, 39 strains of pasteurized *A. muciniphila* with anti-inflammatory properties were preliminarily screened using an *in vitro* HT-29 cell inflammation model. Subsequently, the *in vivo* efficacy of these strains was evaluated in DSS-induced UC mouse models. It is noteworthy that the 39 strains exhibited considerable variation in their efficacy in treating UC. Among them, pasteurized AKK2645 demonstrated the strongest therapeutic efficacy in both *in vitro* and *in vivo*. Pasteurized AKK2645 altered the ratio of Th17/Treg and Th1/Th2 cells in the colon, thereby reducing the mRNA expression of pro-inflammatory cytokines such as *il-6*, *il-1β*, *tnf-α*, *il-17a*, *ifn-γ*, and *il-23*, while simultaneously upregulating the mRNA expression of anti-inflammatory cytokines such as *il-10*, *foxp3*, and *gata3*. Furthermore, 16S rRNA analysis revealed that pasteurized AKK2645 reshaped the gut microbiota composition by increasing the abundance of beneficial bacterial genera such as *Dubosiella*, *Bifidobacterium*, and *Limosilactobacillus*, while simultaneously reducing the abundance of potentially pathogenic genera such as *Ileibacterium* and *Alistipes*. This study provides novel evidence that *A. muciniphila* inactivation exerts strain-specific effects on inflammation regulation. Furthermore, it indicates that pasteurized AKK2645 exerts a beneficial effect on colitis by modulating immune cell populations and microbial homeostasis.

## Graphical abstract

**Figure:**
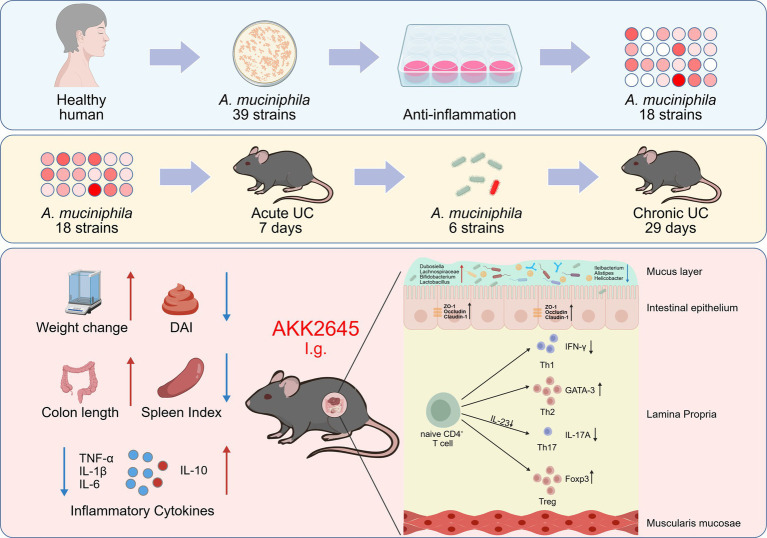
The overall mechanism of pasteurized AKK2645 to mitigate UC (the graphic was created with BioGDP.com).

## 1 Introduction

Ulcerative colitis (UC) is a chronic inflammatory bowel disease characterized by abdominal pain, diarrhea, and bloody stools, imposing a significant morbidity and socioeconomic burden (1, 2). Its global incidence is increasing, driven by factors such as ‘Westernization’ (3). Pathogenesis involves genetic susceptibility, gut dysbiosis, and dysregulated mucosal immunity, which breaks tolerance to commensal bacteria (4, 5). While initially associated with an aberrant Th2 response (6, 7), complex CD4^+^T cell dysfunction is now recognized as central. Notably, the IL-23/Th17 axis is a primary pathogenic driver. Clinical studies consistently report elevated serum IL-17A and IL-23 levels in UC patients, correlating with disease activity (8, 9). A meta-analysis in treatment-naive patients documented median serum IL-23 at 1,167 pg/mL and IL-17A at 1,365.1 pg/mL, compared to control levels of 484.8 pg/mL and 371.5 pg/mL, respectively, establishing this axis as an early hallmark (10). Pathogenic Th17 cells persist following mucosal healing, suggesting their role in relapse (11). Moreover, an elevated Th17/Treg ratio is associated with disease severity and relapse risk (12, 13). Th17 cells therefore constitute a critical nexus in UC pathophysiology, linking acute inflammation to recurrence.

Current UC treatments rely on 5-aminosalicylic acid, glucocorticoids, immunosuppressants and biologics, yet face limitations in efficacy, adverse effects and cost (7). Some patients exhibit primary non-response or secondary loss of response to biologics, with long-term use increasing infection risk (14), underscoring the need for safer, more effective, and cost-efficient strategies. Gut microbiota interventions have emerged as promising alternatives. UC patients exhibit significant dysbiosis. Fecal microbiota transplantation achieves clinical remission in ~30% of patients (15), and probiotics like *Escherichia coli* Nissle 1917 are as effective as mesalazine in maintaining remission (16). *Akkermansia muciniphila* garners attention for its muco-regulatory and immunomodulatory properties, contributing to mucus layer homeostasis (17). Its abundance is significantly reduced in UC patients (active UC: 0.15 ± 0.03% vs. healthy: 0.35 ± 0.05%) (18). The role of live *A. muciniphila* is contentious. While beneficial in many DSS-induced colitis models (19, 20), excessive colonization in a defective mucus layer (e.g., in Muc2^−/−^ mice) may exacerbate inflammation and barrier damage (21). Safety data in immunocompromised populations is also limited (22). Inactivated *A. muciniphila* presents distinct advantages. Pasteurization retains intact bacterial bodies and recapitulates most functions of live bacteria, sometimes with superior efficacy, as demonstrated in metabolic disorder models (23). In DSS-induced colitis, pasteurized *A. muciniphila* reduced disease activity and pro-inflammatory cytokines (24). It selectively modulates gut microbiota, enriching beneficial taxa, reducing pathogens, and enhancing SCFA, particularly butyrate, promoting homeostasis (25). Crucially, it eliminates risks associated with live bacteria, such as colonization, excessive mucus degradation, and bacteremia, while offering improved stability and security (26).

While intra-species functional heterogeneity is a well-established principle in probiotic research (27, 28), existing studies on pasteurized *A. muciniphila* postbiotics have largely focused on single or a few model strains, only nominally acknowledging the possibility of strain-dependent efficacy without systematic empirical validation. Critically, the majority of evidence demonstrating the therapeutic potential of inactivated *A. muciniphila* for UC has been derived from reference strains such as ATCC BAA-835 (29, 30), thereby obscuring the true scope of inter-strain functional variation. From a microbiological perspective, strains of the same species can exhibit substantial differences in genome composition, metabolic characteristics, and immunomodulatory capabilities (31, 32). This raises a fundamental and clinically consequential question: do phylogenetically distinct *A. muciniphila* strains exhibit significant, reproducible functional heterogeneity in alleviating colitis following pasteurization? Here, we fill this critical gap by systematically evaluating the functional differences among pasteurized *A. muciniphila* strains, providing the first empirical demonstration that strain-level heterogeneity dictates postbiotic immunomodulatory efficacy in UC, and establishing a paradigm for strain selection to optimize therapeutic outcomes.

In this study, 39 *A. muciniphila* strains were isolated from the feces of healthy individuals and their anti-inflammatory potential was systematically evaluated after pasteurization using in cellular inflammation models, as well as mouse models of UC. A comprehensive strain library was systematically screened, thereby unveiling strain-dependent disparities in the colitis-alleviating function of pasteurized *A. muciniphila*. One strain, designated pasteurized AKK2645 (previously named YGMCC2645), was identified as a postbiotic candidate with outstanding immunomodulatory potential, providing a theoretical basis and microbial resources for developing novel UC intervention strategies.

## 2 Materials and methods

### 2.1 Bacterial isolation and culture

*A. muciniphila* strains were isolated from fecal samples of healthy human in China. Briefly, 1 g of fecal sample was added to 9 mL of PBS buffer (Solarbio, China) and vortexed thoroughly, followed by 10-fold serial dilution. Aliquots (0.5 mL) from the 10^−4^, 10^−5^, and 10^−6^ dilutions were transferred into 4.5 mL of mucin-containing enrichment medium (Sigma-Aldrich, USA) and incubated anaerobically at 37 °C for 72 h. After incubation, the cultures were serially diluted with PBS, and 0.1 mL of each dilution from 10^−3^ to 10^−6^ was spread onto BHI agar plates (Oxoid, UK). The plates were incubated at 37 °C in an anaerobic incubator (BAKER, UK) for 72 h. Single colonies were picked and purified on BHI agar plates at least three times until colony morphology became uniform. The isolates were preliminarily identified by MALDI-TOF MS (EXS2000, Zybio, China) and subsequently confirmed as *A. muciniphila* by 16S rRNA gene sequencing (Sangon, China). For activation, *A. muciniphila* strains were cultured in modified BHI liquid medium under anaerobic conditions at 37 °C for 36 h to reach an OD₆₀₀ of 0.6, followed by centrifugation at 8,000×*g* for 5 min and washing three times with PBS. The cultures were then pasteurized at 70 °C for 30 min, and complete inactivation was verified by spreading 100 μL of the pasteurized suspension onto BHI agar plates followed by anaerobic incubation for 72 h with no growth observed. The bacterial concentration was then adjusted to 1 × 10^9^ TFU/mL (Total Fluorescence Unit), a unit of total bacterial count determined by fluorescence-based counting (33), and the pasteurized preparations were stored at −80 °C for subsequent experiments.

### 2.2 Cell culture and anti-inflammatory evaluation

Human colorectal adenocarcinoma HT-29 cells (Procell, China) were cultured in MEM medium (Gibco, USA) supplemented with 10% fetal bovine serum (Gibco, USA) at 37 °C in a 5% CO_2_ incubator (Thermo Fisher Scientific, USA). Before the experiment, cells were seeded into 12-well plates at a density of 5 × 10^5^ cells per well and cultured for 24 h to form a monolayer. The experiment included a control group, a model group (LPS + TNF-α), and various strain treatment groups, with four replicate wells per group and three independent experiments. In the treatment groups, cells were incubated with pasteurized *A. muciniphila* at a multiplicity of infection (MOI) of 100 for 6 h. Subsequently, the model and treatment groups were stimulated with LPS (1 μg/mL; Solarbio, China) and TNF-α (10 ng/mL; Solarbio, China), while the control group received an equal volume of medium. After further incubation for 2 h, cell culture supernatants were collected, and IL-8 protein levels were measured using a high-capacity, fully-automated bench-top chemiluminescence immunoassay system (MQ60 PLUS, Hotgen, China).

### 2.3 Animal experiments

#### 2.3.1 Acute UC mouse model

All experimental operations were approved by the Experimental Animal Welfare Ethics Committee of Beijing Sungen Biomedical Technology Co., Ltd. (IACUC-SG-AM-YU-23003). One hundred and twenty eight-week-old male C57BL/6J mice (Charles River, China) were acclimated for 7 days in a standard animal room (temperature: 25 °C ± 2 °C, humidity: 50% ± 5%, 12 h light/dark cycle) and then randomly divided into three groups using the random number table method: Con group, DSS group, and intervention groups (*n* = 6 per group). Mice were housed three per cage. The sample size (*n* = 6 per group) was determined based on established conventions from our research group and previously published studies evaluating treatment efficacy in DSS-induced colitis models (34).

Except for the Con group, all other groups were given free access to drinking water containing 3.0% DSS (MP Biomedicals, USA) in 0.5% sucrose solution for seven consecutive days to induce acute UC. Mice in the intervention groups received daily oral gavage of 0.1 mL of the corresponding pasteurized *A. muciniphila* preparation at a concentration of 1 × 10^9^ TFU/mL, while the Con and DSS groups received an equal volume of PBS. To minimize potential confounders, all cages were randomly arranged on the rack throughout the experimental period, and all assays and measurements were performed in a randomized order.

During the experimental period, body weight, stool consistency, and fecal occult blood (fecal occult blood test kit, Mlbio, China) were monitored daily, and the disease activity index (DAI) was calculated (35). Clear humane endpoints were established during the experiment: any mouse exhibiting a body weight loss exceeding 30% of its initial body weight, or showing signs of severe lethargy or inability to eat/drink (a moribund state), would be immediately removed from the experiment and humanely euthanized. On day 7, the mice were euthanized by carbon dioxide (CO₂) inhalation, and the colon and spleen tissues were collected for measurement of colon length and spleen weight.

Mice that experienced unexpected severe injury unrelated to DSS treatment (e.g., fight wounds) would have been excluded from the analysis. In this experiment, all mice remained healthy throughout the study and no such events occurred; therefore, all mice were included in the final analysis and no data points were excluded. Throughout the experiment, no mice met the aforementioned humane endpoint criteria, and no unexpected adverse events occurred.

#### 2.3.2 Chronic UC mouse model

All experimental operations were approved by the Experimental Animal Welfare Ethics Committee of Beijing Sungen Biomedical Technology Co., Ltd. (IACUC-SG-AM-YU-23003). To evaluate the effects of six pasteurized *A. muciniphila* strains that had shown improvement in the acute UC phenotype, a cyclic DSS-induced protocol was used. Forty-eight eight-week-old male C57BL/6J mice were acclimated in a standard animal room (temperature: 25 °C ± 2 °C, humidity: 50% ± 5%, 12 h light/dark cycle) and then randomly divided into three groups using the random number table method: Con group, DSS group, and intervention groups (*n* = 6 per group). Mice were housed three per cage. The sample size (*n* = 6 per group) was determined with reference to conventional practices in our research group.

Mice in the DSS and intervention groups were given free access to drinking water containing 3% DSS in 0.5% sucrose solution for 5 days, followed by 7 days of 0.5% sucrose solution without DSS. This constituted one cycle, and a total of 2.5 cycles were performed, resulting in a total experimental period of 29 days. Starting from the first day of the experiment, mice in the intervention groups received daily oral gavage of 0.1 mL of pasteurized bacterial preparation at 1 × 10^9^ TFU/mL, while the Con and DSS groups received an equal volume of PBS. To minimize potential confounders, all cages were randomly arranged on the rack throughout the experimental period, and all assays and measurements were performed in a randomized order.

Body weight, stool consistency, and fecal occult blood were monitored, and DAI scores were calculated. Fresh fecal samples were collected from each group 24 h before the end of the experiment. Clear humane endpoints were established during the experiment: any mouse exhibiting a body weight loss exceeding 30% of its initial body weight, or showing signs of severe lethargy or inability to eat/drink (a moribund state), would be immediately removed from the experiment and humanely euthanized. On day 29, the mice were euthanized by carbon dioxide (CO₂) inhalation, and tissues were collected for measurement of colon length and spleen weight.

Mice that experienced unexpected severe injury unrelated to DSS treatment (e.g., fight wounds) would have been excluded from the analysis. In this experiment, all mice remained healthy throughout the study and no such events occurred; therefore, all mice were included in the final analysis and no data points were excluded. Throughout the experiment, no mice met the aforementioned humane endpoint criteria, and no unexpected adverse events occurred.

### 2.4 Tissue staining and scoring

Colon tissues from mice were fixed in 4% paraformaldehyde (Biosharp, China), dehydrated, cleared, embedded in paraffin, and sectioned into 5 μm thick slices. Hematoxylin and eosin (H&E) staining and periodic acid-Schiff (PAS) staining were performed by Wuhan Servicebio Technology Co., Ltd.

All stained sections were observed under a light microscope (Nikon, Japan) to evaluate histopathological changes. H&E staining was used to assess mucosal epithelial structure, inflammatory cell infiltration, crypt architecture, and submucosal edema. PAS staining was used to evaluate the number of goblet cells and mucus secretion. Pathological scoring was performed blindly based on the extent of colonic tissue damage, including depth of inflammatory infiltration, extent of crypt destruction, involvement of the muscularis mucosae, and degree of goblet cell depletion. UC colonic pathological scoring criteria: Inflammatory infiltration: zero points for no infiltration, one point for cumulative mucosal layer, two points for cumulative mucosa and submucosa, and three points for cumulative transmural layer. Tissue damage: zero points for no damage, one point for focal epithelial damage, two points for mucosal erosion and ulcers, and three points for extensive damage to the deep intestinal wall. Crypt destruction: zero points for no destruction, one point for 1/3 range destruction, two points for 2/3 range destruction, three points for 100% crypt destruction, four points for total crypt and intestinal epithelial destruction. Overall lesion range: zero points for lesions in the 0% range, one point for lesions in the 1%–25% range, two points for lesions in the 25%–50% range, three points for lesions in the 50%–75% range, and four points for lesions in the 75%–100% range.

### 2.5 Protein quantification

Colon tissue (0.1 g) was accurately weighed and homogenized in an appropriate volume of PBS using an automatic tissue homogenizer (Jingxin, China). The homogenate was centrifuged at 10,000×*g* for 10 min at 4 °C, and the supernatant was collected. Total protein concentration was determined using a BCA kit (Beyotime, China). The protein levels of TNF-α and IL-6 in the supernatants were measured using ELISA kits (Mlbio, China) strictly according to the manufacturer’s instructions. Standard and sample wells were set up, and the OD_450_ was measured using a microplate reader (Bio-Rad, USA). Cytokine concentrations in each sample were calculated using standard curves.

### 2.6 qPCR

Total RNA was extracted from colon tissues using an RNA extraction kit (TaKaRa, Japan), and its concentration and purity were determined. RNA was reverse-transcribed into cDNA according to the instructions of the reverse transcription kit (TaKaRa, Japan). Using the cDNA as template, qPCR was performed with a SYBR Green qPCR kit (Tiangen, China) on a real-time PCR system (Roche, Switzerland). The reaction program was: 95 °C for 30 s for initial denaturation, followed by 40 cycles of 95 °C for 5 s and 60 °C for 30 s for annealing/extension. GAPDH was used as the internal reference gene, and relative expression levels of target genes were calculated using the 2^−∆∆CT^ method. Primer sequences for the target genes were synthesized by Sangon Biotech Co., Ltd. (China) and are listed in Supplementary Table 4.

### 2.7 Flow cytometric

Fresh colon tissues were dissected to remove mesentery and fat, opened longitudinally, cleaned, and cut into small pieces. The tissues were sequentially treated with pre-digestion buffer and digestion buffer containing an enzyme mix, and dissociation was performed using a gentle tissue dissociator (GentleMACS, Miltenyi Biotec, Germany). After Percoll density gradient centrifugation (Cytiva, USA), mononuclear cells from the colonic lamina propria were isolated. The obtained cells were stimulated for 5 h in 1640 medium (Gibco, USA) containing the cell stimulant GolgiPlug (BD Biosciences, USA). Subsequently, cells were stained with surface antibodies (CD45, CD3, CD4, CD8, etc., all from BioLegend, USA) and intracellular cytokine antibodies (IFN-γ, IL-17A, etc., all from BioLegend, USA). Stained cells were analyzed using a flow cytometer (BD Biosciences, USA), and the proportions and numbers of T cell subsets, including Th1 (CD4^+^ IFN-γ^+^), Th17 (CD4^+^ IL-17A^+^), Th2 (CD4^+^ GATA3^+^), and Treg (CD4^+^ Foxp3^+^) cells, were statistically analyzed using FlowJo software (Tree Star, USA).

### 2.8 16S rRNA gene sequencing analysis

Fecal samples were collected from mice, and total microbial genomic DNA was extracted using a stool DNA extraction kit (Omega Bio-tek, USA) according to the manufacturer’s instructions. DNA samples were amplified using the universal primers 338F and 806R for the V3-V4 variable region of the bacterial 16S rRNA gene (forward primer: 50-ACTCCTACGGGAGGCAGCAG-30; reverse primer: 50-GGAC-TACHVGGGTWTCTAAT-30. ACTCCTACGGGAGGCAGCAG-30; reverse primer: 50-GGACTACHVGGGTWTCTAAT-30). The separation of the PCR products was conducted via 2% agarose gel electrophoresis, followed by purification through utilization of a DNA gel extraction kit (PCR Clean-Up Kit, YuHua, China). The purified library was then subjected to paired-end sequencing on an Illumina NextSeq 2000 platform (Illumina, USA) at Shanghai Majorbio Bio-Pharm Technology Co., Ltd. The raw sequencing data underwent a series of quality control measures, including filtration, trimming, merging, and subsequent OTU clustering and taxonomic analysis. The raw sequencing data have been deposited in the NCBI Sequence Read Archive under BioProject accession number PRJNA1475974. In accordance with the findings of the OTU analysis, alpha diversity indices (e.g., Sobs index, Shannon index) and beta diversity were calculated. Furthermore, differences in gut microbiota composition among groups at various taxonomic levels (e.g., genus level) were analyzed.

### 2.9 Statistical analysis

All statistical analyses were performed using GraphPad Prism 10.1.2 software (GraphPad Software, Inc., La Jolla, CA, USA). Experimental design included 3–6 biological replicates per group, and data are presented as mean ± standard deviation (SD). Prior to group difference analysis, the Shapiro–Wilk test was used to assess the normality of data distribution. For data that met the assumption of normality, one-way analysis of variance (ANOVA) followed by Dunnett’s multiple comparisons test was used. For data that did not meet the assumption of normality, the Kruskal–Wallis *H* test was employed, with Mann–Whitney *U* test or Wilcoxon signed-rank test used as appropriate. Correlation between variables was evaluated using the Spearman rank correlation coefficient. The statistical significance threshold was set at *p* < 0.05.

## 3 Results

### 3.1 Pasteurized *A. muciniphila* alleviating systemic physiological impairments in acute colitis

**Figure 1:**
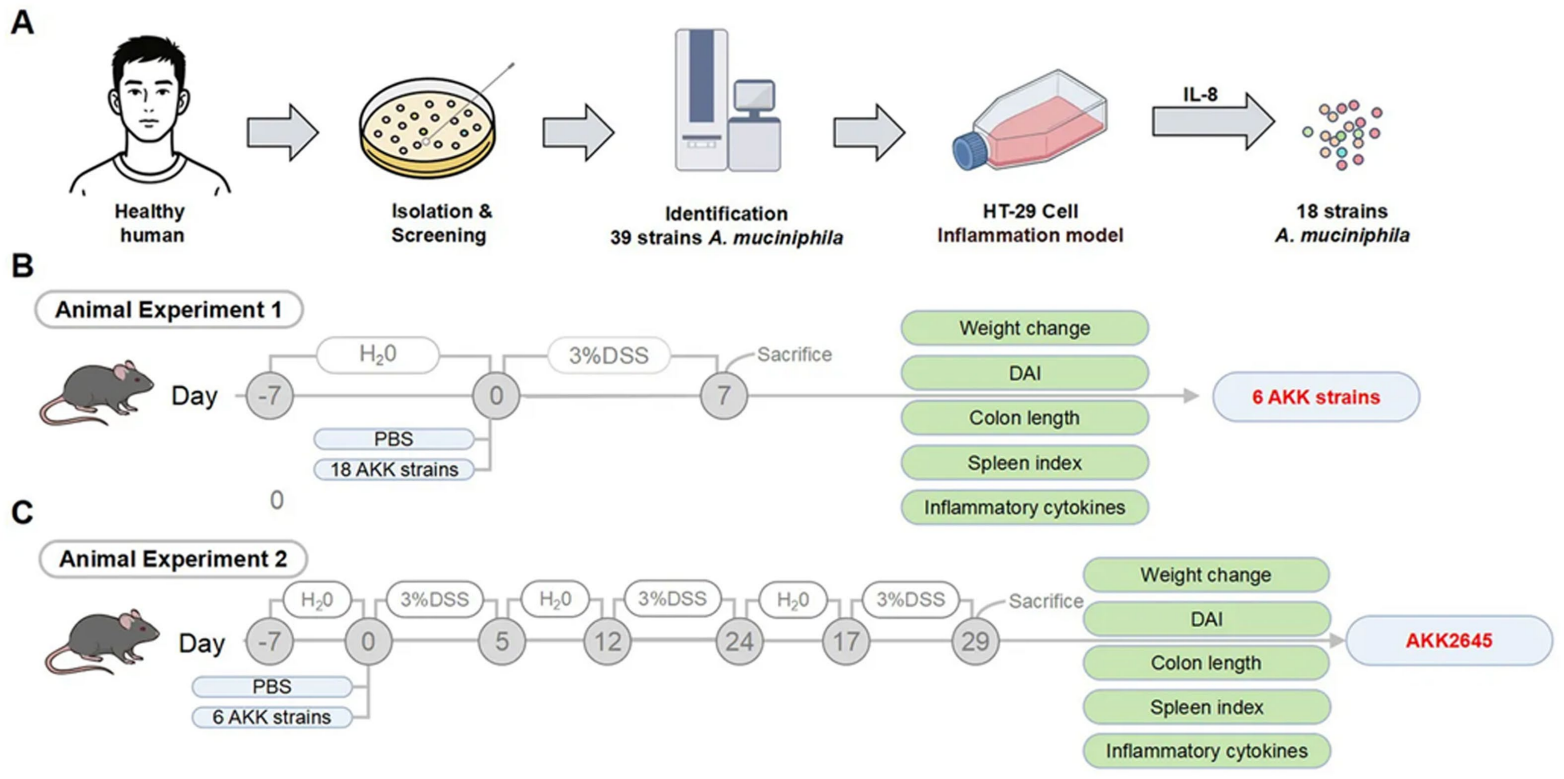
Experimental workflow. **(A)** Schematic diagram of *A. muciniphila* isolation, identification, and pasteurized *A. muciniphila in vitro* anti-inflammatory screening. **(B)** Experimental design for evaluating pasteurized *A. muciniphila* in a 7-day acute UC mouse model. **(C)** Experimental design for evaluating pasteurized *A. muciniphila* in a 29-day chronic UC mouse model.

**Figure 2:**
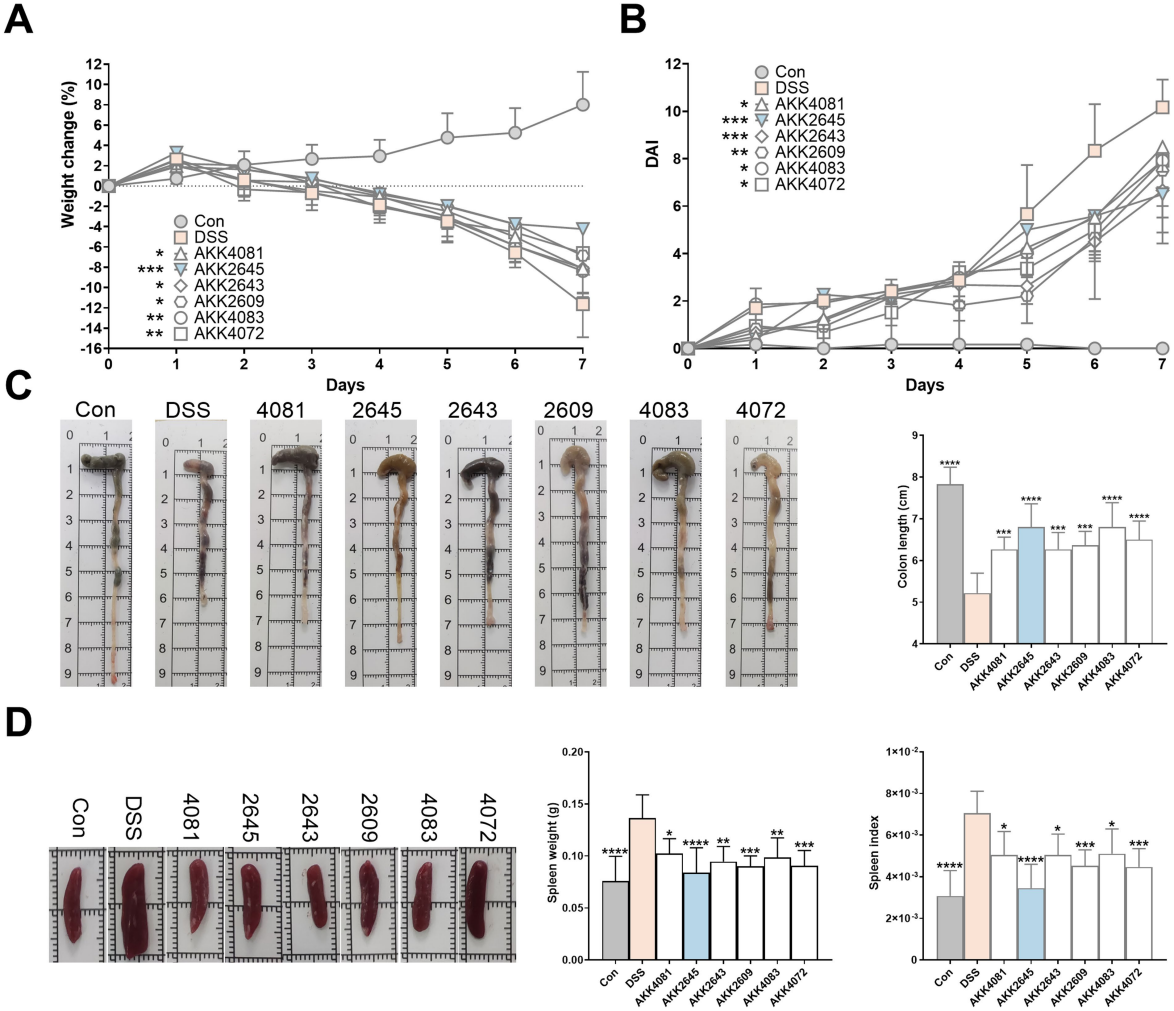
Pasteurized *A. muciniphila* ameliorates the phenotype of DSS-induced acute UC in mice. **(A,B)** Percentage of body weight change and DAI scores. **(C)** Representative images of colons (left) and quantitative colon length (right). **(D)** Representative images of spleens (left), spleen weight (middle), and spleen index (right). Data are presented as mean ± SD; *n* = 6 mice per group. Statistical significance was analyzed by one-way ANOVA followed by Dunnett’s multiple comparisons test versus the DSS group: *p < 0.05, **p < 0.01, ***p < 0.001, ****p < 0.0001.

The inflammatory response is a hallmark of the pathogenesis of UC, and anti-inflammatory activity serves as a critical biomarker for evaluating therapeutic efficacy. The *in vitro* anti-inflammatory potential of 39 pasteurized *A. muciniphila* strains were systematically screened (Figure 1A). Using the inhibition rate of the reference strain ATCC BAA-835 (42.04%) as the selection threshold, a total of 18 pasteurized *A. muciniphila* strains exhibited higher IL-8 inhibition rates in the HT-29 cell inflammation model (Table 1 and Supplementary Table 1). Among these, the highest inhibition rate was observed for AKK4081 (83.31%), while AKK2645 showed an inhibition rate of 62.31%, also meeting the selection criterion and being included for subsequent *in vivo* evaluation. In acute UC model was established in mice using 3% DSS for 7 days (Figure 1B). The DAI score is a recognized standard for the assessment of colitis severity. Mice receiving DSS for 7 days exhibited a significant reduction in body weight and elevated DAI scores. Among the 18 pasteurized strains under test, AKK4081, AKK2643, AKK2609, AKK2645, AKK4083, and AKK4072 notably ameliorated the DSS-induced weight loss and DAI scores (Figures 2A,B). In contrast, the remaining 12 strains did not exhibit significant therapeutic effects (Supplementary Tables 1, 2). Furthermore, all six effective strains significantly ameliorated DSS-induced colon shortening (Figures 2C). Elevated immune organ weight and index are typically associated with inflammatory progression. The DSS group exhibited significantly increased spleen weight and spleen index, which were significantly reduced following intervention with each of the six strains (Figures 2D).

**Table 1:** Inhibition rates of IL-8 by 18 strains of *A. muciniphila* in the HT-29 cell inflammation model.

Strain ID	Inhibition rate (%)
AKK4083	72.89 ± 9.79
AKK4081	83.31 ± 5.21
AKK2616	70.27 ± 3.53
AKK2609	70.12 ± 4.30
AKK4087	68.98 ± 5.87
AKK2639	67.56 ± 1.98
AKK2630	63.86 ± 3.73
AKK2643	62.40 ± 0.75
AKK2645	62.31 ± 4.91
AKK2602	59.51 ± 5.21
AKK2618	57.88 ± 0.61
AKK4071	54.48 ± 7.83
AKK2599	53.86 ± 1.74
AKK2587	52.38 ± 2.25
AKK2594	47.37 ± 3.14
AKK2586	47.13 ± 3.54
AKK2592	42.75 ± 4.00
AKK4072	42.10 ± 6.90
ATCC BAA-835	42.04 ± 5.01

Inhibition rates are presented as the mean ± standard deviation (SD). All experiments were performed in triplicate (*n* = 3 per group).

### 3.2 Pasteurized *A. muciniphila* suppressing colonic proinflammatory cytokines and tissue injury in acute colitis

**Figure 3:**
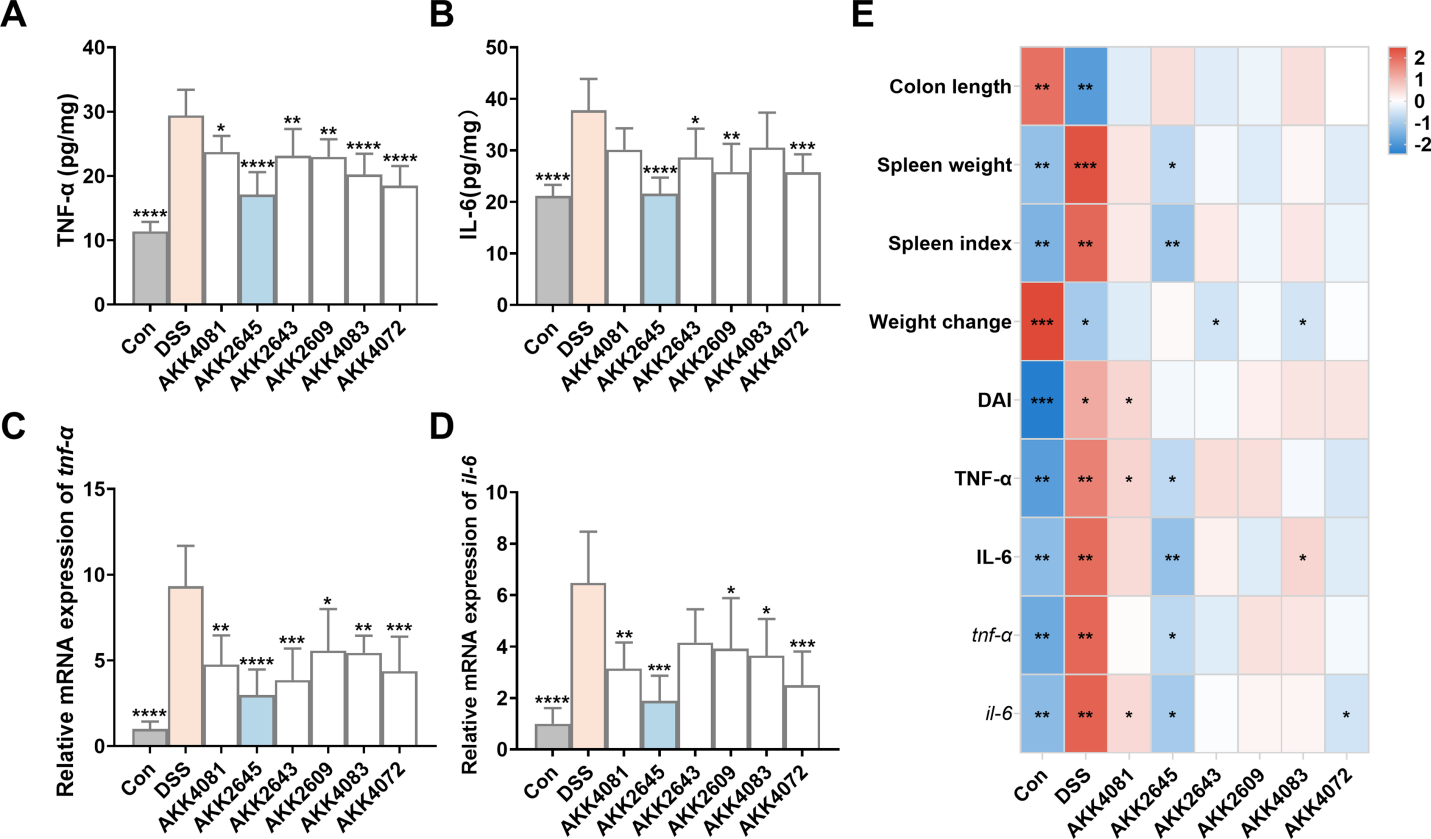
Effects of pasteurized *A. muciniphila* on colonic inflammatory mediators in DSS-induced acute UC mice. **(A,B)** Protein levels of TNF-α and IL-6 in colon tissues. (**C,D**) mRNA expression levels of *tnf-α* and *il-6* in colon tissues. **(E)** Heatmap correlation analysis between each group and UC-related phenotypes/inflammatory markers. Red indicates positive correlation, blue indicates negative correlation, and color intensity reflects the strength of correlation. Data are presented as mean ± SD; *n* = 6 mice per group. Statistical significance was analyzed by one-way ANOVA followed by Dunnett’s multiple comparisons test versus the DSS group: *p < 0.05, **p < 0.01, ***p < 0.001, ****p < 0.0001.

The subsequent phase of the study involved the quantification of pro-inflammatory cytokines IL-6 and TNF-α, in order to evaluate colonic inflammatory damage. In comparison with the control group, the DSS group exhibited significantly elevated levels of IL-6 and TNF-α. It is noteworthy that all six strains were capable of inhibiting TNF-α secretion (Figure 3A). However, only pasteurized AKK2645, pasteurized AKK2643, pasteurized AKK2609, and pasteurized AKK4072 displayed a significant capacity to inhibit IL-6 secretion (Figure 3B). qPCR analysis of *il-6* and *tnf-α* mRNA expression in colonic tissues revealed a marked increase in expression in the DSS group compared with the control group. This increase was only partially reversed by certain strains (Figures 3C,D). In order to assess differential efficacy among the six candidate strains, a comprehensive correlation analysis was performed between each group and colitis-related biomarkers (Figure 3E). This analysis identified distinct strains with varying capacities to ameliorate acute UC phenotypes and inflammation. It is noteworthy that pasteurized AKK2645 exhibited the most significant therapeutic effects, suggesting its potential as a promising postbiotic candidate.

### 3.3 Pasteurized AKK2645 conferring sustained protection against pathological damage and inflammation in chronic relapsing colitis

**Figure 4:**
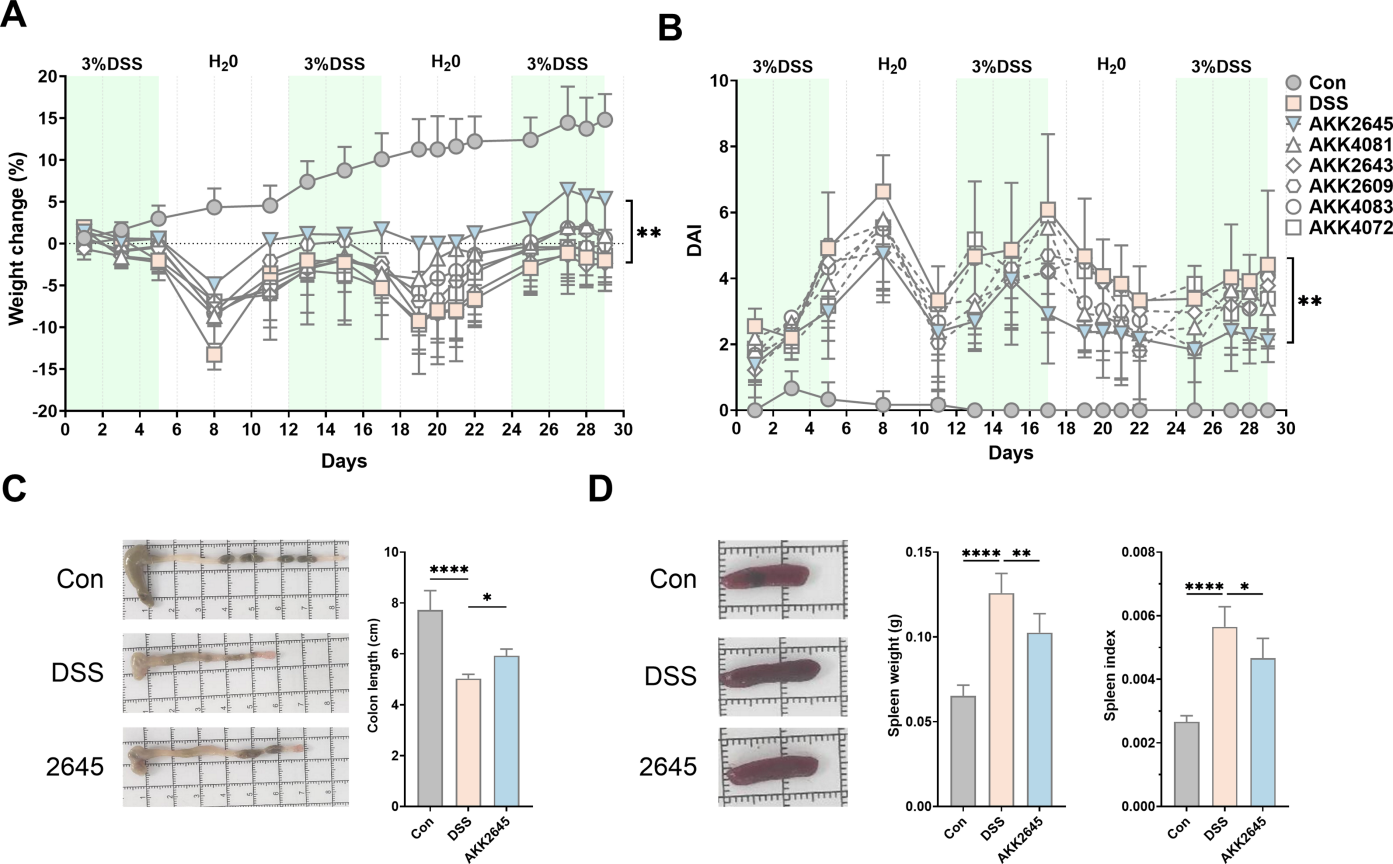
Pasteurized AKK2645 ameliorates the phenotype of chronic UC in mice. **(A)** Percentage of body weight change. **(B)** DAI scores. **(C)** Representative images of colons (left) and quantitative colon length (right). **(D)** Representative images of spleens (left), spleen weight (middle), and spleen index (right). Data are presented as mean ± SD; *n* = 6 mice per group. Statistical significance was analyzed by one-way ANOVA followed by Dunnett’s multiple comparisons test versus the DSS group: *p < 0.05, **p < 0.01, ***p < 0.001, ****p < 0.0001.

In consideration of the discrepancies between the acute model induced by DSS and the chronic immune mechanisms of human UC, it is concluded that this model is only suitable for preliminary screening. The therapeutic efficacy of the model must be validated in chronic mouse models that more closely resemble the pathogenesis of human UC. In order to address this, the present study employed intermittent administration of 3% DSS over 2.5 cycles in order to establish a 29-day chronic UC model (Figure 1C). In the initial cycle, mice in the DSS group exhibited significant weight reduction and heightened DAI scores, which is consistent with the acute UC model. During the remission phase of the first cycle, all six candidate pasteurized strains (AKK2645, AKK4081, AKK2643, AKK2609, AKK4083, and AKK4072) improved DSS-induced weight loss and DAI elevation, with effects consistent with those observed in the acute UC model (Figures 4A,B). However, following the second and third DSS administrations, the ameliorative effects of the five candidate pasteurized strains (with the exception of AKK2645) gradually diminished. Following a 29-day period of DSS intervention, significant reductions in body weight change rates and colonic length were observed in the DSS group, along with a substantial elevation in DAI scores. Among the six candidate strains, only pasteurized AKK2645 intervention significantly improved these pathological manifestations (Figures 4A–C). Moreover, the present study found that pasteurized AKK2645 was effective in alleviating the long-term increases in spleen weight and spleen index induced by DSS exposure (Figure 4D), whereas the other five strains failed to exert a similar protective effect (Supplementary Figures 1A–C).

### 3.4 Analysis of key outer membrane proteins at different *A. muciniphila* strains

**Figure 5:**
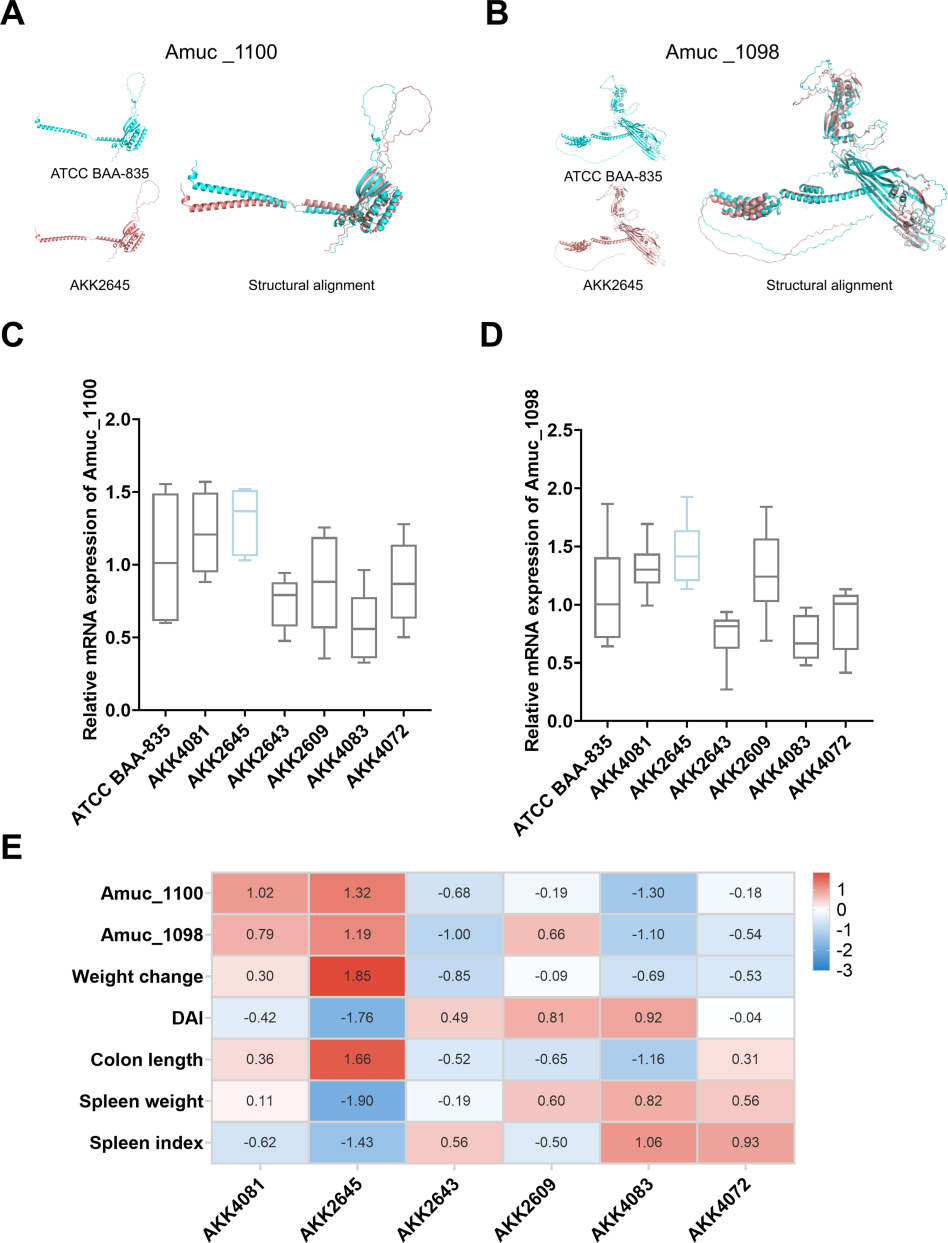
Structural characterization and therapeutic relevance of the outer membrane proteins Amuc_1100 and Amuc_1098 in *A. muciniphila*. **(A,B)** AlphaFold3-predicted structures and PyMOL-based superposition of Amuc_1100 **(A)** and Amuc_1098 **(B)** from AKK2645 (cyan) and ATCC BAA-835 (red). Left panels show individual models; right panels show the superimposed pair. (C, D) qPCR quantification of Amuc_1100 **(C)** and Amuc_1098 **(D)** mRNA relative to ATCC BAA-835 across six pasteurized *A. muciniphila* strains. **(E)** Heatmap of Pearson correlations between Amuc_1100/Amuc_1098 transcript levels and phenotypic indices of chronic UC severity across the six strains. Red, positive correlation; blue, negative correlation. Color intensity denotes correlation strength. Data are presented as mean ± SD; *n* = 6 mice per group. Statistical significance was analyzed by one-way ANOVA followed by Dunnett’s multiple comparisons test versus the DSS group.

The pasteurization protocol employed during *A. muciniphila* strain preparation removes secreted proteins and metabolites, thereby directing the analysis toward outer membrane proteins. Highly conserved and indispensable, Amuc_1100 and Amuc_1098 are outer membrane proteins that orchestrate key immune and metabolic regulatory processes. Across the six test strains, multiple sequence alignments with the reference strain ATCC BAA-835 (performed using SnapGene) indicated that Amuc_1098 nucleotide identity ranged from 99.12% to 99.78% while amino acid identity spanned 99.34%–100%, and Amuc_1100 DNA identity varied from 97.69% to 99.37% with protein identity of 98.42%–99.68% (Supplementary Figures 2A–D). Combining AlphaFold3 (36) structural predictions with PyMOL visualization and superposition further verified that the overall folds of Amuc_1100 and Amuc_1098 from two representative strains (AKK2645 and ATCC BAA-835) are highly congruent, with structural variations largely restricted to surface-exposed loop regions and the conserved α-helical and β-sheet frameworks remaining substantially unchanged (Figures 5A,B). These findings underscore the sequence and structural conservation of Amuc_1100 and Amuc_1098 among the six candidate strains. Nevertheless, transcriptional heterogeneity emerged from qPCR analysis of total RNA isolated from logarithmically growing cultures, revealing that relative mRNA expression of both proteins was higher in AKK2645 than in the other strains and ATCC BAA-835, although the differences did not attain statistical significance (Figures 5C,D). Heatmap visualization further showed that the ability of pasteurized *A. muciniphila* to ameliorate UC correlated with the transcript levels of these two proteins (Figure 5E).

### 3.5 Pasteurized AKK2645 restoring intestinal barrier integrity and ameliorating chronic colonic pathology

Damage to the colonic barrier integrity has been shown to induce inflammatory responses and exacerbate the severity of chronic colitis in mice. The effects of pasteurized AKK2645 on intestinal barrier function were investigated using HE and PAS staining.

**Figure 6:**
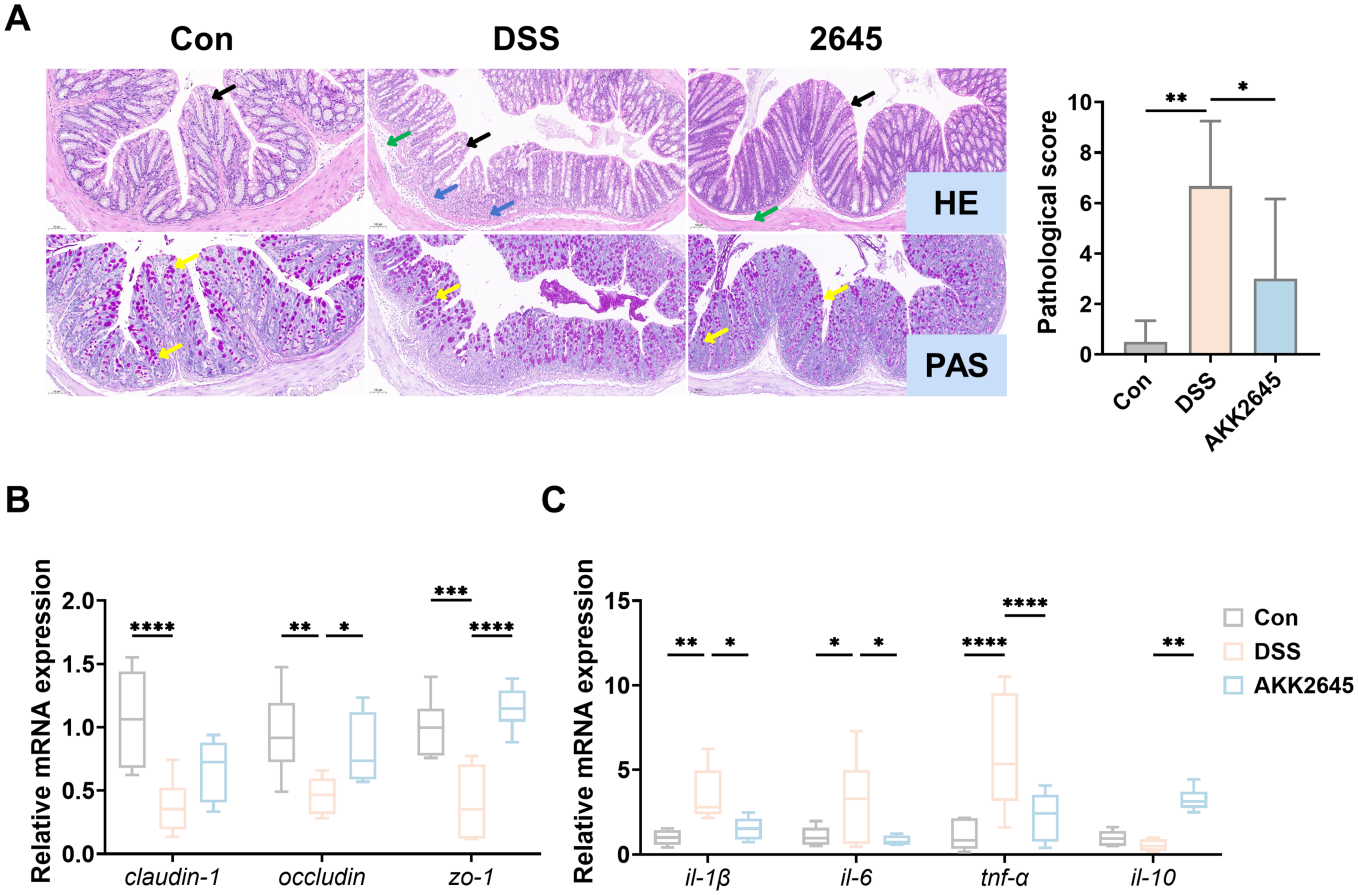
Pasteurized AKK2645 alleviates colonic pathological damage in chronic UC mice. **(A)** Representative micrographs of H&E and PAS-stained colon sections (left) and quantitative histopathological scores (right). Black arrow: colonic mucosal epithelium; blue arrow: inflammatory infiltration; yellow arrow: basal goblet cells; green arrow: submucosal edema. Magnification ×10, scale bar = 100 μm. **(B,C)** mRNA expression levels of inflammatory cytokines and barrier-related genes in colon tissues. Data are presented as mean ± SD; *n* = 6 mice per group. Statistical significance was analyzed by one-way ANOVA (for normally distributed data) or the Kruskal–Wallis test (for non-normally distributed data), followed by Dunnett’s or Dunn’s multiple comparisons test, respectively, versus the DSS group: *p < 0.05, **p < 0.01, ***p < 0.001, ****p < 0.0001.

The results indicated substantial colonic tissue damage in the DSS group, as evidenced by impaired colonic mucosal epithelial structure (black arrow), inflammatory infiltration (blue arrow), reduced basolateral goblet cells (yellow arrow), and submucosal edema (green arrow), accompanied by a significant increase in colonic pathological scores. Conversely, treatment with pasteurized AKK2645 resulted in a significant amelioration of the pathological symptoms and scores (Figure 6A). A subsequent analysis of mRNA expression revealed that, following the DSS intervention, the levels of *zo-1*, *occludin*, and *claudin-1* gene expression were significantly lower than those observed in the control group. However, oral administration of pasteurized AKK2645 significantly enhanced DSS-induced gene expression of *zo-1* and *occludin*, while *claudin-1* expression showed an increase, though not statistically significant (Figure 6B). Furthermore, the repeated administration of DSS in mice resulted in a substantial upregulation of pro-inflammatory cytokines, namely *il-1β*, *il-6*, and *tnf-α*. Concurrent pasteurized AKK2645 intervention significantly suppressed the gene expression of pro-inflammatory factors (Figure 6C). In addition, the impact of pasteurized AKK2645 on the expression of the anti-inflammatory cytokine *il-10* has been evidenced to be significant. The present findings suggest that pasteurized AKK2645 may alleviate DSS-induced chronic colitis by maintaining intestinal barrier function and modulating inflammatory responses.

### 3.6 Pasteurized AKK2645 rebalancing Th1/Th2 and Th17/Treg immune axes in the colonic lamina propria

**Figure 7:**
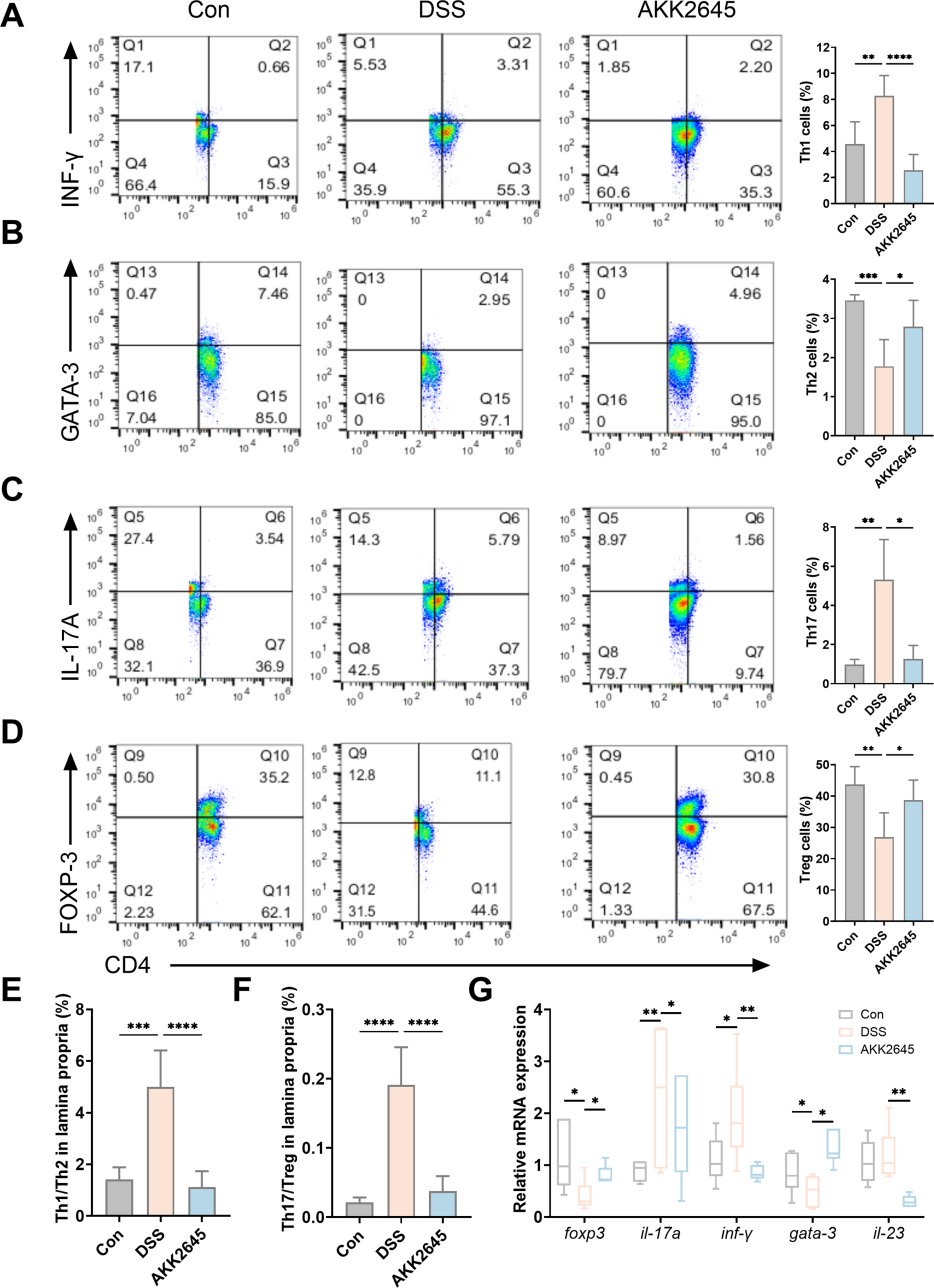
Pasteurized AKK2645 modulates colonic immune cell populations in chronic UC mice. **(A)** Th1 cell counts in the mouse colon. **(B)** Th2 cell counts. **(C)** Th17 cell counts. **(D)** Treg cell counts. **(E,F)** Ratio of Th1/Th2 **(E)** and Th17/Treg **(F)** cells in the mouse lamina propria. **(G)** mRNA expression levels of inflammatory cytokines in colon tissues. Data are presented as mean ± SD; *n* = 6 mice per group. Statistical significance was analyzed by one-way ANOVA (for normally distributed data) or the Kruskal–Wallis test (for non-normally distributed data), followed by Dunnett’s or Dunn’s multiple comparisons test, respectively, versus the DSS group: *p < 0.05, **p < 0.01, ***p < 0.001, ****p < 0.0001.

The homeostasis of Th17/Treg and Th1/Th2 cell ratios is of particular significance in maintaining immune cell balance, preserving mucosal barrier integrity, and regulating inflammation. In the pathogenesis of UC, an immune regulatory imbalance manifests as excessive activation of Th17 cells and functional suppression of Treg cells, leading to massive release of pro-inflammatory factors such as IL-17A and TNF-α, along with enhanced Th1-type immune responses. These processes further compromise intestinal immune tolerance, exacerbating epithelial injury and inflammatory infiltration. In order to address this, a flow cytometry analysis was performed to determine the distribution of immune cell subpopulations in the colonic lymphoid lamina propria of mice. This investigation has established that a DSS intervention can lead to an alteration in Th1/Th2 and Th17/Treg cell ratios in the murine colon. Treatment with pasteurized AKK2645 led to a significant reduction in the numbers of Th1 and Th17 cells, returning them to baseline levels in comparison to the control group. Concurrently, a substantial increase in the numbers of Th2 and Treg cells was observed (Figures 7A–D), thus restoring the equilibrium of Th1/Th2 and Th17/Treg cell ratios (Figures 7E–F). Furthermore, qPCR analysis revealed a decrease in *il-17a* and *inf-γ* gene expression, and a significant increase in *foxp3* and *gata3* mRNA expression following pasteurized AKK2645 treatment (Figure 7G). Furthermore, it was observed that pasteurized AKK2645 led to a significant reduction in the levels of *il-23*, a potent proliferative and differentiation factor for Th17 cells. The results indicated that pasteurized AKK2645 had the capacity to modulate the Th1/Th2 and Th17/Treg cell balance, thus contributing to the alleviation of colonic inflammation in murine models of UC, while concurrently maintaining intestinal immune homeostasis.

### 3.7 Pasteurized AKK2645 remodeling gut microbiota dysbiosis and enriching beneficial commensals

**Figure 8:**
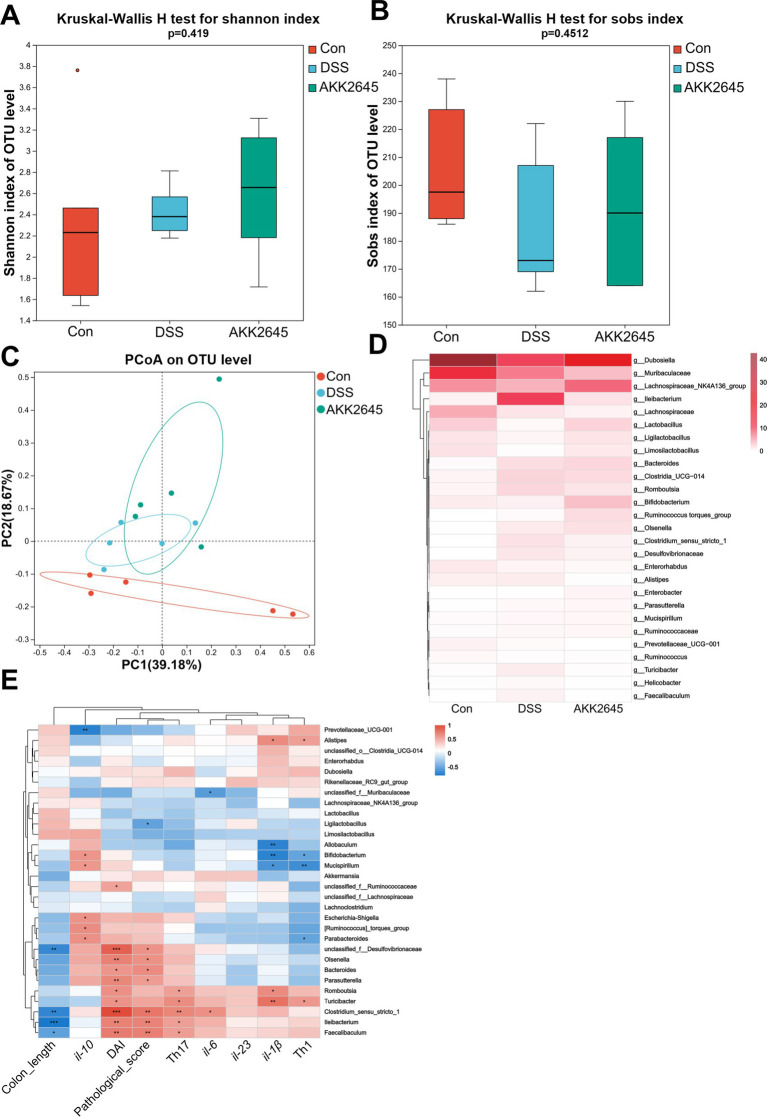
Pasteurized AKK2645 restores the gut microbiota distribution in chronic UC mice. **(A)** Shannon index. **(B)** Sobs index. **(C)** PCoA plot. **(D)** Bacterial community analysis at the genus level. **(E)** Heatmap correlation analysis between gut microbiota and inflammatory markers. Red indicates positive correlation, blue indicates negative correlation, and color intensity reflects the strength of correlation. Both alpha diversity index comparisons and differential analyzes of microbial relative abundance were conducted using the non-parametric Kruskal–Wallis *H* test. Error bars represent SD.

In view of the pivotal function of gut microbiota dysbiosis in UC, postbiotic-targeted modulation of gut microbiota has emerged as a novel strategy for UC improvement. The present study utilized 16S rRNA gene sequencing technology to investigate the hypothesis that pasteurized AKK2645 modulates gut microbiota structure in order to exert its therapeutic effects on UC. The present study employed 16S rRNA gene sequencing to analyze the impact on gut microbiota structure in DSS-induced chronic UC mice. The results showed that the inactivation intervention of pasteurized AKK2645 had a significant effect on DSS-induced gut microbiota dysbiosis. In comparison with the DSS group, pasteurized AKK2645 showed an increase in the Shannon index and a recovery in the Sobs index, but neither difference was statistically significant (*p* > 0.05; Figures 8A,B). In order to further elucidate the role of microbial diversity, β-diversity was analyzed via PCoA. The PCoA plot revealed distinct clustering patterns among the Con, DSS, and AKK2645 groups, indicating significant differences among the three groups. Pasteurized AKK2645 significantly altered the diversity of the gut microbiota (Figure 8C). At the genus level, pasteurized AKK2645 increased the abundance of beneficial bacteria such as *Dubosiella*, *Lachnospiraceae_NK4A136_group*, *Bifidobacterium*, *Lactobacillus*, *Limosilactobacillus*, and *Ligilactobacillus*, while concomitantly reducing the abundance of intestinal pathogenic bacteria including *Ileibacterium*, *Alistipes*, and *Helicobacter* (Figure 8D). In order to further elucidate the potential association between alterations in the composition of the gut microbiota and the various phenotypes associated with UC, correlation analyzes were conducted between the top 30 most abundant bacterial genera identified at the genus level and UC-related physiological indicators (Figure 8E). The results indicated that these beneficial bacteria demonstrated negative correlations with the DAI, pathological scores, pro-inflammatory factors (*il-6*, *il-23*, *il-1β*), and immune cells (Th17 and Th1), as well as positive correlations with colonic length and the anti-inflammatory factor *il-10*. Conversely, an inverse correlation trend was observed between pathogenic bacteria and colitis-related indicators (Figure 7E). In summary, the pasteurized AKK2645 medication has been demonstrated to enhance disease indicators of chronic UC by modulating the diversity and composition of gut microbiota, increasing beneficial bacterial abundance, and reducing pathogenic bacterial abundance.

## 4 Discussion

Systematic screening of 39 pasteurized *A. muciniphila* strains from healthy human in China revealed significant heterogeneity in postbiotic efficacy against UC. A promising candidate, AKK2645, was identified. Evaluation showed strains vary markedly in improving colitis, modulating cytokines, and restoring immune homeostasis, underscoring the need for strain-level comparisons. This aligns with genomic data on strain-specific gene variations (37).

Pasteurized AKK2645 uniquely demonstrates precise, sustained regulation of Th17/Treg and Th1/Th2 balances. While prior studies report that pasteurized *A. muciniphila* regulates cytokines like TNF-α, IL-6, and IL-1β and promotes IL-10 in UC (24, 38), systematic immune cell subset analysis remains limited. Flow cytometric analysis revealed that pasteurized AKK2645 not only suppresses pro-inflammatory Th1 and Th17 cells but also restores anti-inflammatory Th2 and Treg numbers, rebalancing DSS-disrupted ratios. This global remodeling of the UC immune network is unreported for other pasteurized strains. Although inactivated *A. muciniphila* can broadly affect T cell subsets, including modulating CD8^+^ T cells to attenuate colitis-associated tumorigenesis (39), these effects are strain-dependent.

A central empirical finding of this study is that the sustainability of therapeutic benefit in chronic, relapsing inflammatory stress is governed by the functional heterogeneity among *A. muciniphila* strains. While six candidate strains were protective in an acute DSS colitis model, only pasteurized AKK2645 maintained efficacy—evidenced by sustained improvements in body weight, disease activity index, colon length, and spleen index—across repetitive inflammatory cycles in a relapse–remission model. The progressive loss of efficacy observed with the other five strains indicates a deficit in durable immunomodulation, which cannot be attributed to generic postbiotic properties but instead implicates distinct strain-intrinsic molecular determinants.

Given that the pasteurization and preparation process (70 °C, 30 min) includes rigorous washing steps, labile metabolites such as short-chain fatty acids are effectively removed and most secreted soluble proteins are denatured. The observed functional divergence among strains most likely resides in heat-stable cellular architectures rather than diffusible soluble factors. Among the retained surface-associated macromolecules, outer membrane proteins are the most plausible effectors. Amuc_1100 is the prototypical, well-characterized outer-membrane TLR2 immunomodulator of *A. muciniphila* (40). Recent evidence further defines Amuc_1098 as another functionally important surface-anchored membrane protein. Studies show that Amuc_1098 alleviates acute pancreatitis by suppressing NF-κB signaling and remodeling glycerophospholipid metabolism, also via TLR2 engagement (41). Although these two proteins are highly conserved across the species, our data reveal that AKK2645 exhibits the highest relative mRNA expression levels of both Amuc_1100 and Amuc_1098 compared to the other candidate strains. In the context of chronic inflammation, this quantitative difference in transcriptional output likely translates to a higher density of functional effectors on the bacterial surface. As both proteins are capable of engaging TLR2 to restrain inflammatory cascades, the superior capacity of AKK2645 to suppress pathogenic Th17 cells and promote Treg differentiation is likely proportional to the abundance of these surface-exposed immunomodulators.

Strain-specific differences extend beyond direct host effects to include distinct patterns of gut microbiota modulation. Pasteurized AKK2645 selectively promotes the enrichment of *Dubosiella*, a genus may linked to the biosynthesis of the anti-inflammatory sphingolipid sphingosine (42). Sphingosine and related metabolic derivatives inhibit NF-κB signaling, enhance regulatory T cell differentiation, and attenuate pathogenic Th17 responses (43). Consequently, AKK2645 may orchestrate a synergistic, two-pronged mechanism: direct host immunomodulation mediated by strain-specific effector molecules coupled with indirect metabolic reprogramming via cross-feeding interactions that support sphingolipid-producing commensals. This postulated postbiotic–commensal–metabolite amplification cascade would not be replicated by strains devoid of the precise molecular signatures required to facilitate *Dubosiella* proliferation.

It is important to ascertain why pasteurized AKK2645 exhibits such divergent durability of efficacy in acute versus chronic models. The acute UC model (7 days) is proposed to primarily reflect chemical barrier disruption and initial immune responses. During this period, multiple anti-inflammatory signaling pathways (e.g., NF-κB inhibition, upregulation of barrier proteins) can respond rapidly, thus allowing many strains to exert short-term protective effects (44, 45). Conversely, the chronic UC model (2.5 cycles) reproduces the remission-relapse pathological process of human UC, with persistent immune activation and a substantial Th17/Treg imbalance as defining features. The persistence of pathogenic Th17 cells, together with insufficient Treg function, constitutes the basis for relapse (46). Pasteurized AKK2645 has been shown to possess a distinctive immune reprogramming capacity, suppressing Th17 pathogenic conversion and promoting Treg survival and tissue repair. By contrast, other strains may offer only transient, non-specific anti-inflammatory signals that fail to reverse the immune imprinting caused by chronic inflammation.

Further investigation must determine whether live or inactivated *A. muciniphila* is most therapeutic for UC. While both forms show efficacy, pasteurized *A. muciniphila* appears to offer a superior safety profile and mechanistic advantage in UC’s compromised mucosal environment. The metabolic activity of live bacteria could exacerbate mucus erosion, whereas pasteurization retains key immunomodulatory components (e.g., Amuc_1100) while eliminating this risk (23). It also ensures safety against bacteremia and product stability. Efficacy of pasteurized strain AKK2645 in colitis models, restoring immune and microbial balance, supports that an inactivated postbiotic may be a more potent and reliable UC therapy. Future development should prioritize engineered, pasteurized, strain-specific formulations for UC and similar barrier-defect conditions.

Finally, this study has several limitations. First, although the DSS-induced colitis model recapitulates certain pathological features of human UC, it cannot fully replicate the complex immunogenetic background and chronic relapsing course of the human disease. Second, only male mice were used in this study; given that UC does not exhibit a marked sex bias in humans, the generalizability of these findings to female subjects requires further investigation. Finally, while we observed that pasteurized AKK2645 modulates the Th17/Treg balance and gut microbiota composition, the precise molecular mechanisms (e.g., the specific signaling pathways mediated by the Amuc_1100 protein) warrant further elucidation using gene-knockout models and *in vitro* receptor-blocking experiments.

In summary, through large-scale strain screening, this study reveals for the first time significant inter-strain differences in the colitis-alleviating function of pasteurized *A. muciniphila* postbiotics and successfully identifies pasteurized AKK2645 as a candidate strain with outstanding immune-reprogramming capacity. In the context of chronic inflammatory stress, this strain has been observed to exert sustained and stable therapeutic effects by precisely regulating the Th17/Treg balance, repairing the intestinal barrier, and remodeling the gut microbiota (particularly enriching *Dubosiella*). The safety of pasteurized AKK2645 has been fully proven at present (47), and it is the only *A. muciniphila* strain for which the National Health Commission of the People’s Republic of China has accepted a new food raw material application Its future application prospects are very promising. It is recommended that future studies combine comparative genomics, functional molecular characterization (e.g., Amuc_1100 variant analysis) and gene-knockout mouse models in order to thoroughly elucidate the core effector molecules of pasteurized AKK2645 and their interactions with the host and microbiota. This would provide a solid foundation for its clinical translation.

## Data Availability

The 16S rRNA sequencing data presented in this study are deposited in the NCBI Sequence Read Archive (SRA) repository, BioProject accession number PRJNA1475974.
