# Supplementary material for: Strain-dependent efficacy of pasteurized Akkermansia muciniphila in alleviating colitis reveals AKK2645 restores immune and microbial homeostasis
Source: Front Nutr. 2026 Jul 27;13:1863368. doi: 10.3389/fnut.2026.1863368 (PMC13455503; doi:10.3389/fnut.2026.1863368)
Supplement: Supplementary file 1 [file Supplementary_file_1.docx]

**Supplementary Figure 1**. Effects of pasteurized other 5 strains on the phenotype of chronic UC in mice. (A) colon length. (B) spleen weight. (C) spleen index. Data are presented as mean ± SD; n = 6 mice per group. Statistical significance was analyzed by one-way ANOVA followed by Dunnett’s multiple comparisons test versus the DSS group: **p* < 0.05, ***p* < 0.01, ****p* < 0.001, *****p* < 0.0001.

**Supplementary Figure 2.** Sequence alignments of Amuc_1100 and Amuc_1098 using ATCC BAA-835 as the reference strain. (A) Protein sequence alignment of Amuc_1100. (B) Protein sequence alignment of Amuc_1098. (C) DNA sequence alignment of Amuc_1100. (D) Gene sequence alignment of Amuc_1098.

**Supplementary Table 1.** Inhibition rates of IL-8 by the remaining 21 strains of *A. muciniphila* in the HT-29 cell inflammation model.

| **Strain ID** | **Inhibition rate(%)** | **Strain ID** | **Inhibition rate(%)** |
| --- | --- | --- | --- |
| AKK2596 | 40.78 ± 0.63 | AKK2589 | 29.07 ± 8.66 |
| AKK2595 | 40.53 ± 5.17 | AKK2583 | 28.67 ± 1.36 |
| AKK2610 | 39.93 ± 6.02 | AKK2584 | 23.17 ± 11.74 |
| AKK2590 | 39.34 ± 3.90 | AKK2601 | 20.44 ± 1.77 |
| AKK2591 | 35.43 ± 1.77 | AKK2588 | 13.52 ± 3.76 |
| AKK2598 | 35.22 ± 4.75 | AKK2593 | 12.95 ± 4.70 |
| AKK2603 | 34.53 ± 2.54 | AKK2625 | 1.87 ± 6.56 |
| AKK2585 | 32.95 ± 1.51 | AKK2606 | 0.59 ± 8.26 |
| AKK2600 | 31.89 ± 6.31 | AKK2607 | -8.21 ± 10.97 |
| AKK2597 | 30.73 ± 1.79 | AKK2604 | -15.46 ± 11.50 |
|  |  | AKK2605 | -23.35 ± 1.75 |

Inhibition rates are presented as the mean ± standard deviation (SD). All experiments were performed in triplicate (n = 3 per group).

**Supplementary Table 2.** Weight changes(%) of the remaining 12 strains of pasteurized *A. muciniphila* in the 7-day acute UC model.

| **Strain ID** | **Days** | | | | | | |
| --- | --- | --- | --- | --- | --- | --- | --- |
|  | **1** | **2** | **3** | **4** | **5** | **6** | **7** |
| Con | 0.73±1.41 | 2.09±1.33 | 2.67±1.39 | 2.95±1.6 | 4.76±2.41 | 5.25±2.42 | 8.01±3.24 |
| DSS | 2.61±0.97 | 0.59±0.69 | -0.71±0.55 | -1.87±1.02 | -3.47±0.75 | -6.56±1.46 | -10.63±4.4 |
| AKK2602 | 1.90±1.28 | 2.00±1.33 | 1.39±2.52 | 0.74±1.67 | -2.08±2.78 | -5.13±2.79 | -8.80±3.92 |
| AKK2618 | 0.51±1.14 | 0.75±1.74 | -0.01±1.48 | -2.03±1.56 | -4.21±3.13 | -10.26±5.61 | -14.85±7.10 |
| AKK2599 | 0.88±1.33 | 0.86±1.33 | 1.03±1.53 | -0.72±0.99 | -4.02±3.85 | -10.59±4.86 | -15.64±5.27 |
| AKK2594 | 0.19±1.34 | -0.19±1.61 | -1.07±1.16 | -1.17±1.89 | -3.53±3.10 | -6.63±4.10 | -10.50±6.08 |
| AKK2586 | 1.22±0.59 | 1.80±1.48 | 0.57±0.96 | -0.04±1.38 | -2.77±1.79 | -6.42±3.73 | -10.21±5.84 |
| AKK2587 | 0.91±1.71 | 0.74±1.47 | 0.06±1.67 | 0.00±1.73 | -2.51±2.41 | -6.68±4.44 | -10.34±5.22 |
| AKK2592 | 1.72±1.71 | 1.14±1.86 | 0.76±1.65 | -1.59±1.8 | -3.04±3.98 | -10.00±3.97 | -15.27±4.84 |
| AKK4087 | 2.44±2.36 | 1.82±1.68 | 1.40±2.70 | 0.80±2.79 | -3.50±4.11 | -7.88±9.63 | -10.83±7.29 |
| AKK4071 | 2.75±3.47 | 1.78±4.14 | 0.34±4.43 | -1.65±4.49 | -3.87±3.57 | -6.20±4.62 | -9.26±5.27 |
| AKK2639 | 2.55±0.84 | 0.01±1.37 | -0.80±1.93 | -1.26±1.68 | -3.84±3.05 | -7.31±3.24 | -11.19±4.32 |
| AKK2630 | -0.01±1.74 | -0.73±1.56 | -0.09±1.69 | -1.25±1.68 | -5.58±3.27 | -7.57±4.73 | -10.64±5.22 |
| AKK2616 | 0.64±1.32 | 0.03±1.26 | -0.64±1.52 | -1.94±2.05 | -4.27±2.01 | -7.25±1.53 | -11.09±3.61 |

Weight changes are presented as the mean ± standard deviation (SD).Statistical significance compared to the DSS group was determined using one-way ANOVA followed by Dunnett's multiple comparison test. **p* < 0.05 was considered statistically significant.

**Supplementary Table 3.** DAI score of the remaining 12 strains of pasteurized *A. muciniphila* in the 7-day acute UC model.

| **Strain ID** | **Days** | | | | | | |
| --- | --- | --- | --- | --- | --- | --- | --- |
|  | **1** | **2** | **3** | **4** | **5** | **6** | **7** |
| Con | 0.17±0.41 | 0.00±0.00 | 0.17±0.41 | 0.17±0.41 | 0.17±0.41 | 0.00±0.00 | 0.00±0.00 |
| DSS | 1.70±0.83 | 2.02±0.04 | 2.42±0.49 | 2.87±0.78 | 5.67±2.07 | 8.33±1.97 | 10.17±1.17 |
| AKK2602 | 1.40±0.93 | 1.17±1.13 | 0.42±0.78 | 3.27±2.45 | 5.83±1.94 | 7.58±0.92 | 8.33±1.86 |
| AKK2618 | 1.47±0.91 | 1.27±1.14 | 1.45±1.40 | 4.83±1.94 | 7.33±1.97 | 9.33±2.36 | 10.50±1.76 |
| AKK2599 | 3.50±2.35 | 2.20±1.48 | 1.80±1.15 | 4.96±2.62 | 7.50±2.08 | 10.25±1.71 | 11.50±0.58 |
| AKK2594 | 1.32±0.97 | 1.33±0.82 | 0.77±0.86 | 2.83±1.97 | 5.17±1.72 | 8.67±2.07 | 9.67±1.97 |
| AKK2586 | 1.57±0.92 | 1.50±1.73 | 1.15±0.66 | 4.00±0.63 | 6.42±1.74 | 7.92±2.06 | 9.50±1.87 |
| AKK2587 | 1.72±1.19 | 1.70±1.38 | 1.28±0.79 | 3.17±1.58 | 6.83±1.72 | 8.33±1.89 | 8.83±1.83 |
| AKK2592 | 1.30±0.99 | 1.45±1.07 | 1.87±1.15 | 4.92±1.50 | 6.58±0.92 | 8.83±2.48 | 9.75±2.04 |
| AKK4087 | 0.68±1.06 | 0.60±1.27 | 2.72±0.94 | 3.35±1.31 | 6.70±3.94 | 7.92±3.64 | 10.40±1.14 |
| AKK4071 | 1.12±1.17 | 1.18±1.34 | 1.70±1.20 | 2.90±0.89 | 6.00±1.61 | 7.55±2.25 | 8.03±2.55 |
| AKK2639 | 1.33±1.03 | 1.17±1.33 | 1.67±1.34 | 2.13±1.35 | 4.92±1.20 | 8.25±2.19 | 9.83±1.72 |
| AKK2630 | 0.95±0.99 | 2.08±0.57 | 1.98±0.68 | 2.88±1.25 | 6.33±2.48 | 7.58±2.52 | 9.63±3.15 |
| AKK2616 | 1.58±1.20 | 1.22±1.28 | 2.50±1.23 | 2.52±1.04 | 5.25±1.57 | 7.25±2.19 | 10.67±1.47 |

DAI score was presented as the mean ± standard deviation (SD). Statistical significance compared to the DSS group was determined using one-way ANOVA followed by Dunnett's multiple comparison test (n = 6 mice per group). **p* < 0.05 was considered statistically significant.DAI scores were calculated as the combined score of weight loss, stool consistency, and gross bleeding.

| Primers | Forward | Reverse |
| --- | --- | --- |
| *gaphd* | AGGTCGGTGTGAACGGATTTG | GGGGTCGTTGATGGCAACA |
| *tnf-α* | CATCTTCTCAAAATTCGAGTGACAA | TGGGAGTAGACAAGGTACAACCC |
| *il-1β* | CAGGCAGGCAGTATCACTCAT TG | CGTCACACACCAGCAGGTTA TC |
| *il-10* | GCTCTTACTGACTGGCATGAG | CGCAGCTCTAGGAGCATGTG |
| *il-6* | CCAGTTGCCTTCTTGGGACT | GGTCTGTTGGGAGTGGTATCC |
| *zo-1* | GACCTTGAGCAGCCGTCATA | CCGTAGGCGATGGTCATAGTT |
| *occludin* | CAGCCTTCTGCTTCATCG | GTCGGGTTCACTCCCATTA |
| *claudin1* | CATCGGCAGCAGCATCATCAC | ACGATGGTGATCTTGGCCTTGGρ |
| *gata3* | AGGATGTCCCTGCTCTCCTT | GCCTGCGGACTCTACCATAA |
| *il-23* | AGCGGGACATATGAATCTACTAAGAGA | GTCCTAGTAGGGAGGTGTGAAGTTG |
| *foxp3* | CCCATCCCCAGGAGTCTTG | ACCATTGGTTTACTCGCATGT |
| *il-17a* | CTGGAGGATAACACTGTGAGAGT | TGCTGAATGGCGACGGAGTTC |
| *ifn-γ* | TTTTCAGCTCTGCATCGTTTTGGGT | CCTTGAAACAGCATCTGACTCCTT |
| *Amuc_1100* | AAATAGCCTTCCAGGGCGAC | TGCGGATACGGATGGAGTTG |
| *Amuc_1098* | ACTGGATTGTCACCCCGTTC | CAGCAGATTGTCCACGGAGT |
| *A. muciniphila* | AGTCCTGGCAGTAAACGGTG | TCCGAAGAGTCGCATGCTTT |

**Supplementary Table 4.** Sequences of primers used for quantitative real-time PCR (qPCR) analysis.
